# Near-infrared photothermal therapy using anti-EGFR-gold nanorod conjugates for triple negative breast cancer

**DOI:** 10.18632/oncotarget.21243

**Published:** 2017-09-23

**Authors:** Meihua Zhang, Hoe Suk Kim, Tiefeng Jin, Jisu Woo, Yin Ji Piao, Woo Kyung Moon

**Affiliations:** ^1^ Department of Radiology, Seoul National University Hospital, Jongno-gu, Seoul 03080, Korea; ^2^ Department of Biomedical Sciences, Seoul National University College of Medicine, Jongno-gu, Seoul 110-799, Korea; ^3^ Department of Radiology, Yanbian University Hospital, JiLin Province 133000, China; ^4^ Department of Pathology and Cancer Research Center, Yanbian University Medical College, Yanji 133002, China

**Keywords:** near-infrared photothermal therapy, photoacoustic imaging, breast cancer, gold nanorod, epidermal growth factor receptor

## Abstract

Current EGFR-targeted therapy for triple negative breast cancer (TNBC) has produced disappointing results. A rational therapeutic strategy to improve EGFR-targeted treatment for TNBC is therefore needed. In this study we evaluated the feasibility of treating TNBC using photoacoustic imaging (PAI)-guided near-infrared photothermal therapy (NIR-PTT) with anti-EGFR-conjugated gold nanorods (anti-EGFR-GN). NIR-PTT combined with anti-EGFR-GN exerted synergistic anti-proliferative and apoptotic actions through upregulation of HSP70 and cleaved caspase-3, downregulation of Ki-67 and EGFR, and inhibition of several intracellular signaling molecules (mTOR, AKT, ERK1/2 and FAK). These combined effects give this approach significant efficacy. Our findings suggest PAI-guided NIR-PTT using anti-EGFR-GN represent a novel and effective strategy for EGFR-targeted therapy in TNBC.

## INTRODUCTION

Although triple negative breast cancer (TNBC) is a small percentage of all breast cancers, TNBC is one of the most challenging types of breast cancer for basic and clinical research because TNBC patients display a high risk of relapse, shorter overall survival and limited therapeutic options after completion of conventional chemotherapy compared with patients with other breast cancer subtypes [1, 2]. Because of the lack of a well-defined clinical therapy strategy, advances in the design of strategies for the treatment of TNBC require further elucidation, by combined targeted therapy, of the molecular mechanisms underlying TNBC genotypic and phenotypic heterogeneity. Approximately 70 to 80% of TNBCs more frequently overexpress epidermal growth factor receptor (EGFR), which is emerging as a therapeutic target [3, 4]. Many clinical studies on TNBC patients using an EGFR antibody (cetuximab) and EGFR pathway inhibitors (lapatinib and gefitinib) have been evaluated, yet EGFR-targeted therapy has produced a response in only a minority of TNBC patients [5, 6]. A rational therapeutic strategy to overcome the limitations of current EGFR-targeted treatments of TNBC patients is absolutely required for future clinical trials.

Non-invasive monitoring of tumors *in vivo* with biocompatible contrast agents including specific targeting molecules after multiple rounds of intravenous administration may be capable of being optimized and an efficacious treatment of cancer. Photoacoustic imaging (PAI), which can create multi-contrast images of living biological structures ranging from organelles to organs, has shown great translational potential from bench to bedside due to its inexpensiveness and convenience for combination with clinical ultrasound (US) [7]. Gold nanorods (GN) conjugated with specific molecules including antibodies has been proposed as an attractive PAI contrast agent for allowing the GN to bind selectively to certain primary tumors and metastatic sites *in vivo* [8–10]. GN with particularly near-infrared (NIR) optical properties at wavelengths from 700 to 1000 nm, where NIR radiation is able to penetrate the skin without damaging normal tissues, has revealed great potential for simultaneously combining selective targeted imaging and NIR-mediated photothermal therapy (NIR-PTT) in diverse types of cancer [11–14]. In recent years, NIR-PTT using GN embedded within tumors can cause apoptotic or necrotic damage to tumor cells by inducing a localized hyperthermia effect, suggesting a promising therapeutic technique with great potential for cancer treatment due to its minimal invasiveness and high spatial selectivity [15–21]. To date, GN heated with an NIR light has improved the efficiency and safety of therapy against various solid tumors, yet few studies have demonstrated the successful treatment of TNBC.

We previously applied US and PAI using GN conjugated with anti-EGFR antibody (anti-EGFR-GN) for the selective visualization of EGFR-positive TNBCs and demonstrated that US-guided PAI can sensitively detect solid primary tumors as well as lymph node (LN) micrometastases in xenograft mice intravenously injected with anti-EGFR-GN [22]. Anti-EGFR-GN with a desired NIR wavelength (approximately 808 nm) is an ideal contrast agent for application of both cancer cell imaging and NIR-PTT. In the present study, we evaluated the feasibility of using anti-EGFR-GN combined with NIR-PTT for more effective EGFR-targeted therapy of TNBCs with the help of non-invasive monitoring of selective targeting as well as therapeutic response using US and PAI.

## RESULTS

### Anti-EGFR-GN selectively uptaken by EGFR-overexpressing cells, and the combination of anti-EGFR-GN and NIR-PTT synergistically induces cell death

**Figure 1:**
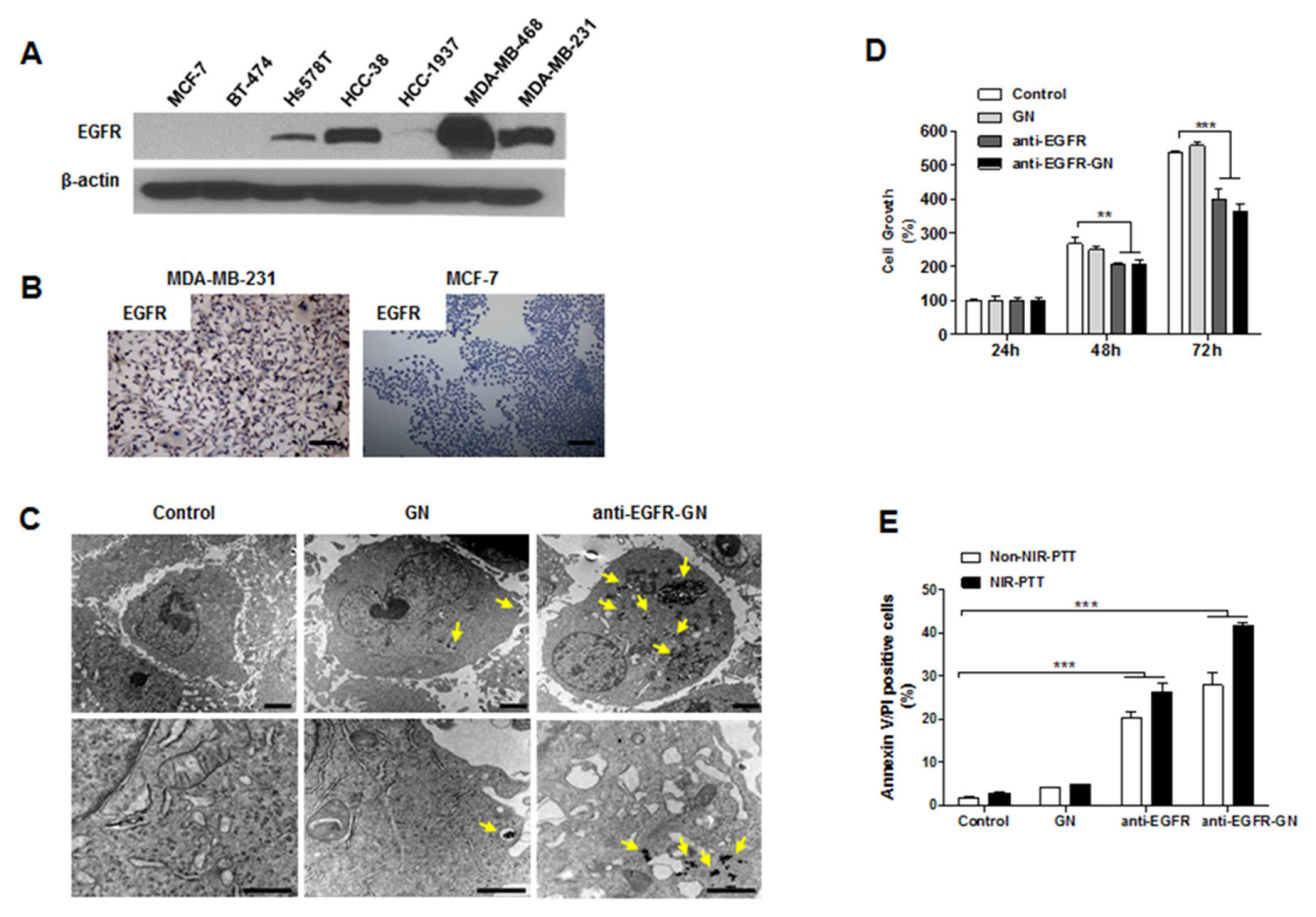
Analysis of selective uptake of anti-EGFR-GN, anti-proliferative and apoptotic action caused by anti-EGFR-GN combined NIR-PTT **(A)** Western blot of EGFR in the total lysates of TNBC cell lines (Hs578T, HCC-38, HCC-1937, MDA-MB-468, MDA-MB-231), ER+ cell line (MCF-7) and HER2+ cell line (BT-474). **(B)** Immunocytochemistry for EGFR in MDA-MB-231 cells and MCF-7 cells. Scale bar: 300 μm. **(C)** TEM images of MDA-MB-231 cells treated with 1.84 μg/ml of GN or anti-EGFR-GN (endocytic uptake, yellow arrows) for 24 h. Scale bar: 1 μm. **(D)** Analysis of cell growth (mean±S.E., n=5) assessed by MTT assay in MDA-MB-231 cells treated with 1.84 μg/ml of GN and anti-EGFR-GN or anti-EGFR antibody (0.22 μg/ml) for 24, 48 and 72 h. **(E)** Apoptotic cell death (mean±S.E., n=4) assessed by flow cytometric analysis of annexin V and PI in MDA-MB-231 cells treated with GN (1.84 μg/ml) and anti-EGFR-GN (1.84 μg/ml GN, 0.22 μg/ml anti-EGFR antibody) or anti-EGFR antibody (0.22 μg/ml) for 24 h and subsequent NIR-PTT for 3 min. *P<0.05, **P < 0.01, ***P < 0.001.

High EGFR expression was detected in TNBC cell lines (Hs578T, HCC-38, MDA-MB-468 and MDA-MB-231), but not in the other subtype cell lines (MCF-7 and BT474) (Figure 1A). Immunocytochemistry also indicated high EGFR expression in MDA-MB-231 cells (Figure 1B). Silver staining showed that the uptake of anti-EGFR-GN by MDA-MB-231 cells overexpressing EGFR was inhibited by competition with free anti-EGFR antibody, indicating the specificity of EGFR-targeting (Supplementary Figure 1). Consistent with the understanding that nanoparticles are endocytosed by cells [10], TEM images revealed a large amount of anti-EGFR-GN in the endosomes or lysosomes of MDA-MB-231 cells. Whereas, low cytoplasmic GN was rarely observed (Figure 1C). 24 h or 72 h treatment with anti-EGFR-GN (208.50±6.78% or 364.31±11.13%, respectively) or anti-EGFR (207.53±1.48% or 339.20±17.14%, respectively) significantly inhibited the cell growth versus untreated control (*P*<0.01 or *P*<0.001), but GN did not affect the cell growth (Figure 1D). Additionally, we observed an anti-cancer effect of anti-EGFR-GNs in another TNBC cell line, MDA-MB-468. Anti-EGFR-GNs suppressed the growth of MDA-MB-468 cells and exhibited similar anti-cancer efficacies observed in MDA-MB-231 cells (Supplementary Figure 2). *In vitro* NIR-PTT (1.5 W/cm^2^, for 3 min) with anti-EGFR-GN can elevate the temperature of cell culture media from to 39°C to 43°C. In flow cytometric analysis of annexin V/propidium iodide (PI) (Figure 1E), a significant apoptotic cell death was not detected in groups treated with NIR-PTT (2.66±0.11%), GN (3.91±0.17%), and GN+NIR-PTT (4.46±0.16%) compared with control (1.74±0.12%). However, treatment with anti-EGFR (20.10±0.81%), anti-EGFR-GN (27.81±1.75%), a combination of anti-EGFR and NIR-PTT (26.46±1.16%) and a combination of anti-EGFR-GN and NIR-PTT (41.51±0.54%) induced significant apoptotic cell death (*P*<0.001). The combined anti-EGFR-GN and NIR-PTT led to an increase in therapeutic temperature to 43°C, consequently resulting in the most effective apoptotic death.

### Anti-EGFR-GN combined with NIR-PTT augmented anti-proliferative and cell death signaling

**Figure 2:**
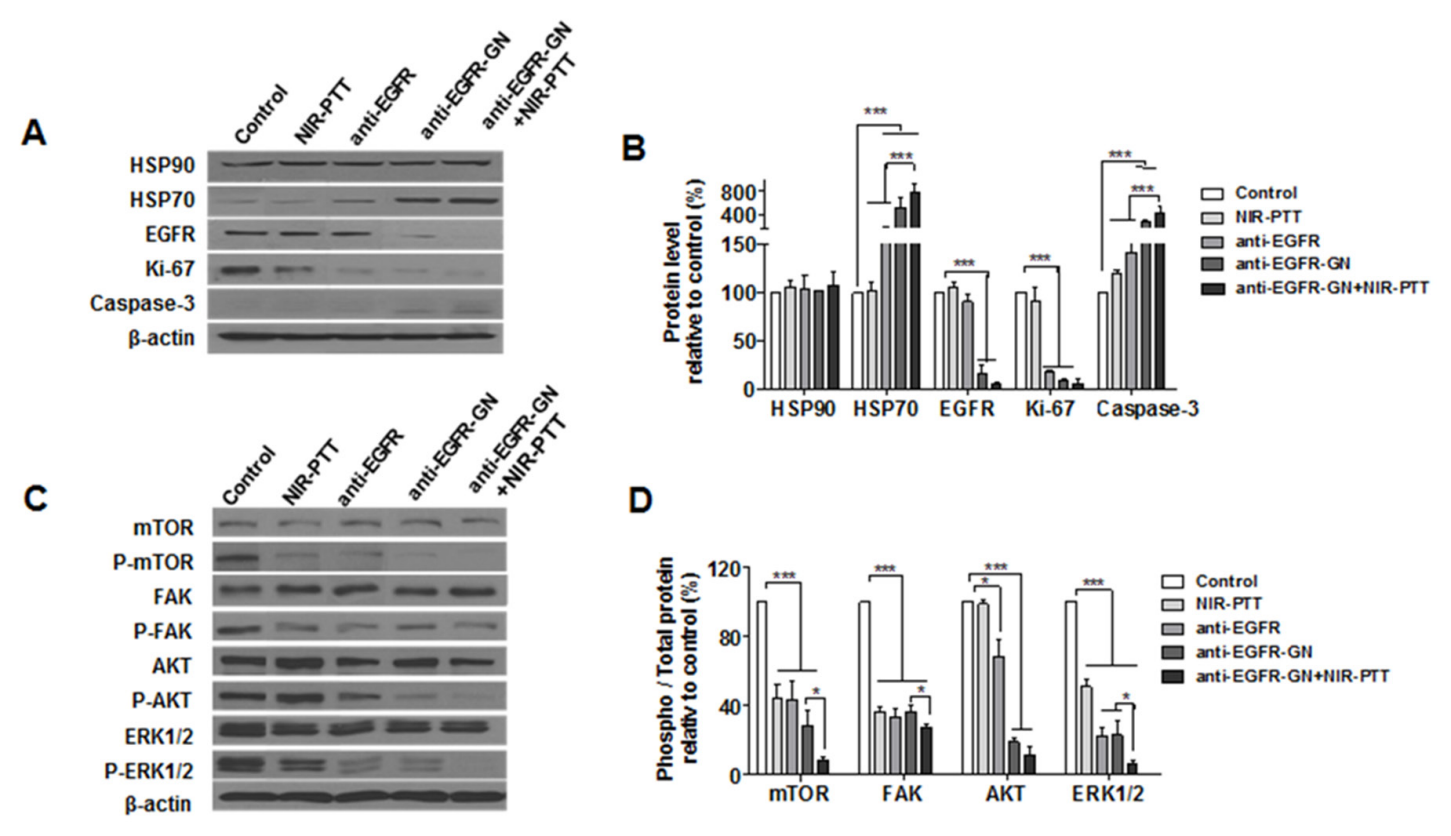
Analysis of the fundamental mechanisms of anti-proliferation and apoptosis triggered by anti-EGFR-GN combined with NIR-PTT **(A** and **C)** Western blot of heat shock proteins (HSP90, HSP70), EGFR, Ki-67, cleaved caspase-3 and EGFR-mediated intracellular signaling molecules (mTOR, FAK, AKT, ERK1/2) in MDA-MB-231 cells treated with GN (1.84 μg/ml), anti-EGFR-GN (1.84 μg/ml GN, 0.22 μg/ml anti-EGFR-antibody) and anti-EGFR antibody (0.22 μg/ml) for 24 h and subsequent NIR-PTT for 3 min. **(B** and **D)** Data (mean±S.E., n=3) obtained from western blot experiments. **P < 0.01, ***P < 0.001.

In this study, GN and GN+NIR-PTT groups were excluded because the cellular uptake of GN and GN+NIR-PTT-induced apoptosis was not observed in MDA-MB-231 cells. We next investigated the effect of anti-EGFR, anti-EGFR-GN and anti-EGFR-GN+NIR-PTT on the alteration of intracellular proteins involved in proliferative and apoptotic activity. As shown in Figure 2A and 2B, there was no difference in HSP90 levels among all groups. Whereas, induction of HSP70 was obvious in groups treated with anti-EGFR (151.53±15.59%), anti-EGFR-GN (520.43±90.87%) and anti-EGFR-GN+NIR-PTT (782.14±80.74%) relative to contro1 (*P*<0.001). The proliferation marker Ki-67 was significantly decreased in groups treated with anti-EGFR (17.1±1.37%), anti-EGFR-GN (9.06±0.60%), and anti-EGFR-GN+NIR-PTT (6.19±2.5%) relative to control (*P*<0.001). The apoptotic marker cleaved caspase-3 was significantly increased in groups treated with anti-EGFR (141.11±11.10%), anti-EGFR-GN (259.08±17.23%), and anti-EGFR-GN+NIR-PTT (422.33±61.36%) relative to control. The other apoptotic biomarker, cleaved poly(ADP-ribose)polymerase-1 (PARP-1) was also evaluated. Cells treated with anti-EGFR-GN+NIR-PTT, although an accumulation of cleaved PARP-1 was not significant as cleaved caspase-3 result, expressed comparable high cleaved PARP-1 (218.10±49.31%) as compared with those of NIR-PTT, GN, GN+NIR-PTT and anti-EGFR-GN (Supplementary Figure 3). A significant decrease of EGFR was evident in the groups treated with anti-EGFR-GN (16.49±4.64%) and anti-EGFR-GN+NIR-PTT (5.89±1.16%) relative to control (*P*<0.001). In the experimental condition for short-term treatment (1, 5, 15, 30 min) with GN or anti-EGFR-GN, the treatment with GN alone increased the phosphorylations of AKT and ERK1/2, but the treatment with anti-EGFR-GN alone suppressed the phosphorylations of FAK and AKT (Supplementary Figure 4). As shown in Figure 2C and 2D, phosphorylations of mTOR, FAK and ERK1/2 were significantly decreased in the groups treated with NIR-PTT (43.99±4.94%, 36.54±1.37% and 50.97±2.62%, respectively), anti-EGFR (43.46±6.43%, 33.60±2.59% and 22.64±2.79%, respectively), anti-EGFR-GN (28.51±4.74%, 36.34±2.11% and 23.04±4.44%, respectively) and anti-EGFR-GN+NIR-PTT (8.51±1.11%, 27.67±0.87% and 6.37±1.26%, respectively) relative to control (*P*<0.001). Phosphorylated AKT was significantly decreased in the groups treated with anti-EGFR (68.12±5.89%, *P*<0.01), anti-EGFR-GN (18.83±1.26%, *P*<0.001) and anti-EGFR-GN+NIR-PTT (11.48±2.78%, *P*<0.001) relative to control. Anti-EGFR-GN+NIR-PTT exerted the most potent synergistic, anti-proliferative and cell death effect through the downregulation of Ki-67 and EGFR, the inhibition of mTOR, FAK, AKT and ERK1/2-mediated intracellular signaling pathways and the activation of caspase-3.

### Anti-EGFR-GN combined with NIR-PTT was a more effective tumor treatment than anti-EGFR-antibody therapy

**Figure 3:**
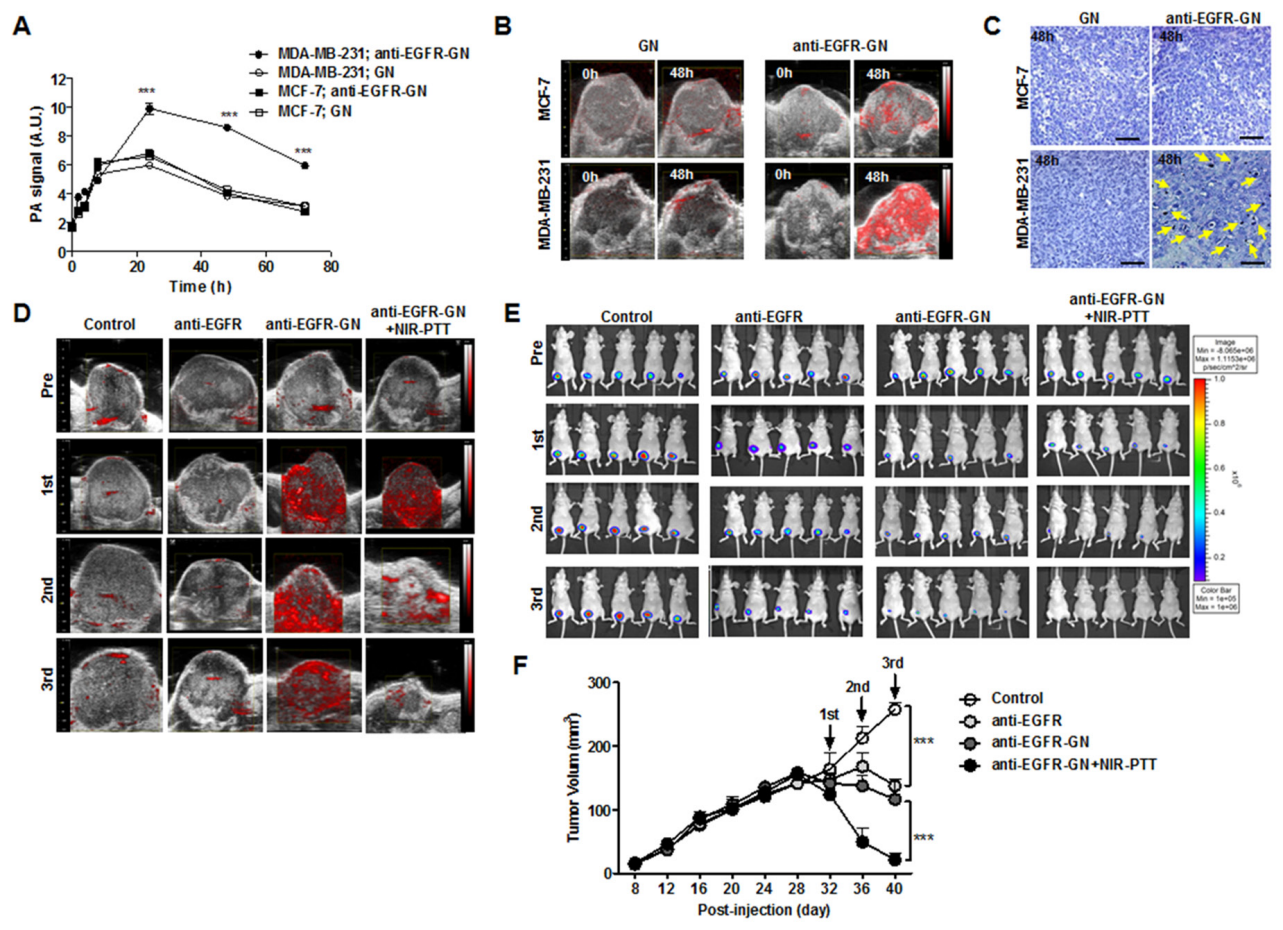
Analysis of PAI-and BLI-guided NIR-PTT combined with anti-EGFR-GN in xenograft tumors **(A)** PA signals (mean±S.E.) measured from MCF-7 and MDA-MB-231 tumors before and 2 h, 4 h, 8 h, 24 h, 48 h and 72 h after intravenous injection with of GN (7.7 mg/kg) or anti-EGFR-GN (7.7 mg/kg GN, 0.92 mg/kg anti-EGFR antibody). **(B)** Representative US and PAI in MCF-7 and MDA-MB-231 tumors before and at 48 h after injection of GN or anti-EGFR-GN. **(C)** Silver staining of MCF-7 and MDA-MB-231 tumors isolated from mice at 48 h post-injection. Yellow arrows indicate the accumulated anti-EGFR-GNs. Scale bar: 200 μm. **(D** and **E)** NIR-PTT was performed at 48 h after injection of anti-EGFR-GN three times at 3-day intervals. Serial follow-up PAI and BLI of tumors in of each group before and after administration of anti-EGFR antibody (0.92 mg/kg), anti-EGFR-GN (7.7 mg/kg) and anti-EGFR-GN (7.7 mg/kg GN, 0.92 mg/kg anti-EGFR antibody)+NIR-PTT. **(F)** Tumor volumes (mean±S.E.) measured from 5 tumors of each group. *P<0.05, **P < 0.01, ***P < 0.001.

The timing of NIR-PTT treatment at which intravenously injected anti-EGFR-GNs maximally accumulated into the tumors of mice using serial follow-up PAI was determined based on our previous study [22]. As shown in Figure 3A, there was no different in PA signal amplitude of GN- and anti-EGFR-GN-treated MCF-7 tumors at each time point. The maximal value of the anti-EGFR-GN-enhanced PA signals (9.91±0.41 AU and 8.59±0.17 AU, respectively) were reached 24 and 48 h post-injection in MDA-MB-231 tumors and was significantly higher (approximately 2-fold) than GN-enhanced PA signals (5.97±0.12 AU and 3.84±0.09 AU, respectively) (Figure 3B, *P*<0.001). GN-enhanced PA signals were decreased into a basal level after 48 h. Figure 3B shows the representative US-guided PAI of MCF-7 and MDA-MB-231 tumors before and at 48 h after intravenous injection with GN or anti-EGFR-GN. Silver staining revealed nanoparticle accumulation in only MDA-MB-231 tumor tissues at 48 h post-injection of anti-EGFR-GN (Figure 3C). These results demonstrate that anti-EGFR-GN selectively accumulates into the MDA-MB-231 tumors, but GN rapidly clears out of the MCF-7 and MDA-MB-231 tumors. In order to effectively achieve EGFR-targeted NIR-PTT for MDA-MB-231 tumor treatment, we excluded the GN+NIR-PTT group from the *in vivo* study and selected an NIR-PTT treatment time of 48 h after anti-EGFR-GN injection. Irradiation with NIR light for 3 min induced mild hyperthermia (43°C) in tumor skin. The longitudinal follow-up US-guided PAI and BLI were acquired from MDA-MB-231 tumors before and after NIR-PTT in each group (Figure 3D and 3E). In the anti-EGFR-GN+NIR-PTT group, the PA signal amplitude and bioluminescence activity of tumors remarkably decreased after second and third treatments, which may selectively reflect EGFR-positive tumor cell damage or tumor vascular insult. Continuous decrease of tumor volumes was observed in the anti-EGFR-, anti-EGFR-GN- and anti-EGFR-GN+NIR-PTT-treated mice versus control (Figure 3F). Anti-EGFR-GN+NIR-PTT resulted in almost complete tumor regression. Treatment with anti-EGFR, anti-EGFR-GN and anti-EGFR-GN+NIT-PTT did not cause significant differences in the body weights of mice and the damage of normal tissues compared to those of untreated mice, suggesting the absence of physical distress over the course of the experiment.

**Figure 4:**
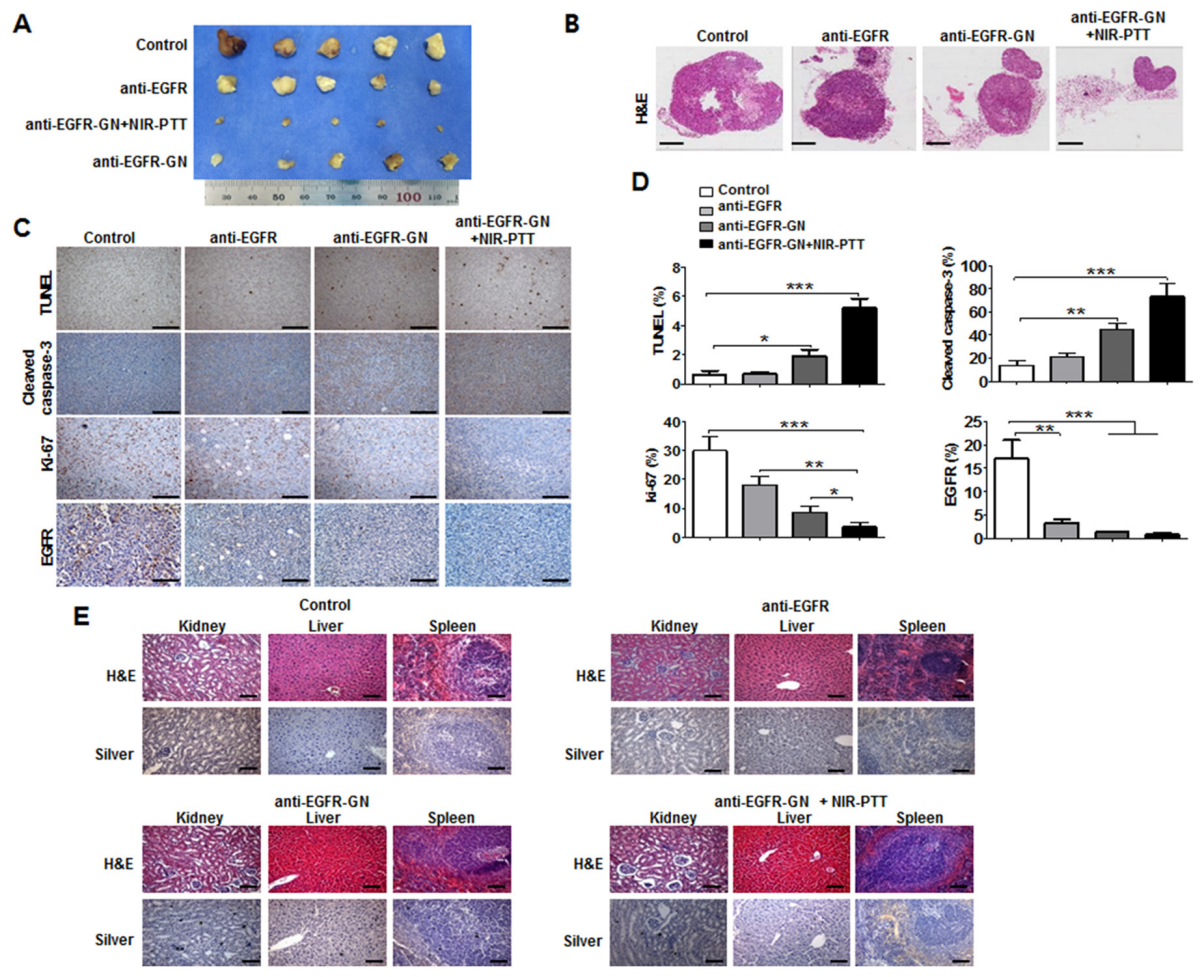
Histological analysis of xenograft tumors Tumor, kidney, liver and spleen was isolated from mice at 4 days after third treatment of anti-EGFR antibody (0.92 mg/kg), anti-EGFR-GN (7.7 mg/kg GN, 0.92 mg/kg anti-EGFR antibody)+NIR-PTT. **(A** and **B)** Gross images and H&E images of tumors isolated from each mouse group. **(C)** Immunohistochemistry images of TUNEL, cleaved caspase-3, Ki-67 and EGFR. **(D)** Quantitative analysis (mean±S.E.) of immunohistochemistry obtained from 5 tumor sections of each group. **(E)** H&E and silver staining of kidney, liver and spleen tissues isolated from each mouse group. Scale bar: 100 μm. *P<0.05, **P < 0.01, ***P < 0.001.

The gross images of tumor tissues revealed that the smallest residual tumor volume was in the anti-EGFR-GN+NIR-PTT group (Figures 4A and 4B). Consistent with the *in vitro* results of cultured cells, immunohistochemical analysis showed much higher caspase-3 (73.33±6.17%) and TUNEL levels (5.2±0.38%) and remarkably lower Ki-67 (3.67±0.88%) and EGFR (0.83±0.20%) levels in the anti-EGFR-GN+NIR-PTT tumors compared with the other tumors (Figures 4C and 4D, *P*<0.001). This indicates that anti-EGFR-GN+NIR-PTT led to the most effective therapy for tumor regression through augmentation of the synergic mechanisms of anti-proliferation and apoptosis. Silver staining revealed very little anti-EGFR-GN accumulation in the kidney at 4 days after the third post-treatment of anti-EGFR-GN+NIR-PTT. H&E staining showed no damage or histological alteration of the liver, spleen or kidney of each group (Figure 4E), implying that anti-EGFR-GN was eventually cleared out of body without toxicity.

## DISCUSSION

We here demonstrate that anti-EGFR-GNs is a useful therapeutic strategy for applying EGFR-targeted NIR-PTT to aggressive TNBC due to their properties of selective tumor cell targeting, long circulation time and desired optical absorption upon NIR irradiation. When combining NIR-PTT with anti-EGFR-GNs in EGFR-positive MDA-MB-231 cells and tumors, we observed obstruction of the EGFR-mediated intracellular signal pathway (low phosphorylated mTOR, AKT, ERK1/2 and FAK levels) involved in cell proliferation and survival, as well as strong anti-proliferative (low Ki-67 level) and apoptotic activity (high cleaved caspase-3 and TUNEL levels).

TNBC is a biologically heterogeneous disease. Translational and clinical studies for emerging targeted TNBC therapies have been facilitated by increased understanding of the aberrant gene expression regulating growth and survival [3, 4]. EGFR represents a clinically relevant molecular target for TNBC patients [6, 23, 24]. Many clinical Phase I/II investigations for TNBC patients have tried using Cetuximab, FDA-approved anti-EGFR antibody [5, 6, 25, 26], yet the results have been somewhat disappointing. With the help of non-invasive longitudinal US-guided PAI, we have previously reported that anti-EGFR-GN has great promise in taking advantage of EGFR-targeted diagnosis [22].

NIR-PTT has gained popularity and has quickly developed in recent years due to minimally invasive treatments for cancer therapy in both fundamental and clinical studies. Considerable research is being directed towards developing GN as a theranostic platform [10, 14, 27]. GN has been brought to the forefront of cancer research in recent years due to convenient surface modifications for specific molecular delivery and tunable optical properties for PAI and NIR-PTT applications [7, 10, 14, 28, 29]. One of the most promising directions in NIR-PTT is the use of GN with 10 to 100 nm in diameter and peak transmission at approximately 800 nm, owing to its long term circulation from an intravenous injection and its high capability of deeper penetration into tissues [7, 8, 15, 29]. Anti-EGFR-GN is an ideal agent for NIR-PTT applications *in vivo* due to its properties of peak absorbance at 808 nm as well as their diameter and length of 10×40 nm. However, to the best of our knowledge, NIR-PTT using anti-EGFR-GN has not yet been applied to TNBCs. Moreover, the detailed biological mechanisms of cellular therapeutic response to anti-EGFR-GN combined with NIR-PTT have not been explored.

A water-filtered infrared A lamp (wIRA), a special form of heat radiation with high tissue penetration and low thermal load to the skin surface, has been applied in wound healing without discomfort to the patient and can be used for cancer therapy [30]. The possible modes of cell death triggered by PTT include necrosis and apoptosis. More specifically, high-energy irradiation can lead to necrosis (>45°C), and low-energy irradiation (<45°C) can promote apoptosis [31, 32]. In our study, the temperature of culture medium and tumor skin increased to 39-43°C after irradiation with wIRA for 3 min.

We found that significant uptake of anti-EGFR-GNs suppressed the proliferative activity of MDA-MB-231 cells compared to that with non-targeted GNs, which is involved in blocking the activation of EGFR-mediated signaling molecules (mTOR, AKT, FAK and ERK1/2) and suppressing EGFR and Ki-67 expressions through the selective and effective delivery of the anti-EGFR-antibodies. In our study, anti-EGFR-GNs combined with NIR-PTT induced an increase in HSP70 and cleaved caspase-3, inhibited the phosphorylations of mTOR, AKT, FAK and ERK1/2 and suppressed EGFR and Ki-67 in cultured MDA-MB-231 cells, which suggests a molecular mechanism of synergistic apoptotic and anti-proliferative activity, not necrosis. In addition, our recent study showed that a combination of anti-EGFR-GN and NIR-PTT induced an autophagic cell death mechanism, resulting in the strongest cell death [33]. Taken together in an *in vitro* study, anti-EGFR-GN combined with NIR-PTT has proven to be a selective and effective therapy in EGFR-expressing TNBC cells. As we expected, anti-EGFR-GNs combined with NIR-PTT led to remarkably greater tumor regression compared to treatment with either anti-EGFR antibody alone or anti-EGFR-GNs alone, significantly reducing proliferation activity (low Ki-67 level) and inducing apoptotic activity (high cleaved caspase-3 and TUNEL levels) in sections of the MDA-MB-231 tumors. Anti-EGFR-GN or a combination of anti-EGFR-GN and NIR-PTT may provide additional therapeutic potential for those TNBC patients overexpressing EGFR who do not respond to anti-EGFR antibody treatment.

In the present study, we suggest that NIR-PTT combined with anti-EGFR-GN is an encouraging therapeutic strategy to improve conventional EGFR-targeted therapy for TNBC patients. However, for the clinical use of anti-EGFR-GNs combined with NIR-PTT, nanoparticle size (5-6 nm) should be reduced in order for the body to more easily clear them [34]. In regards to the clinical use of wIRA, the typical total irradiances are approximately 80-160 mW/cm^2^. For the use of wIRA in the treatment of breast cancer, application of wIRA with appropriate therapeutic irradiation intensities and doses should be considered as being safe depending on the size of the irradiated area, tissue temperature, and the amount of subcutaneous soft tissues.

## MATERIALS AND METHODS

### GN, anti-EGFR-GN and breast cancer cell lines

GN and anti-EGFR-GN (10 nm×40 nm) conjugated with 16 anti-EGFR antibodies per GN were purchased from Nanopartz, Inc. (Loveland, CO, USA). NIR responsive GN and anti-EGFR-GN were synthesized as previously reported [22]. The human breast cancer cell lines were obtained from the Korean Cell Line Bank (Seoul, Korea): estrogen receptor positive (ER+) (MCF-7), human epidermal growth factor receptor 2 amplified (HER2+) (BT-474) and TN (Hs578T, HCC-38, HCC-1937, MDA-MB-453 and MDA-MB-231). MDA-MB-231-Luc cells stably expressing firefly luciferase were established using lentivirus.

### Immunocytochemistry

Cells were fixed in 2% paraformaldehyde and blocked with 2% bovine serum albumin. The cells were incubated with anti-EGFR antibody (Cell Signaling Technology, Danvers, MA, USA), followed by incubation with an appropriate secondary antibody. The proteins were visualized with 3,3-diaminobenzidine, and hematoxylin was used as counterstain. The images were acquired using a microscope equipped with a CCD camera (Leica, Wetzlar, Germany).

### Transmission electron microscopy (TEM)

Cells were fixed with 2.5% glutaraldehyde, treated with 2% osmium tetroxide in 0.1 mmol/L cacodylate buffer for 2 h, dehydrated with ethanol (50 to 100%) and propylene oxide, and embedded in pure Epon resin at 60°C for 3 days. Ultrathin sections were cut with glass knives and a Diatome diamond knife (Reichert-Jung, Vienna, Austria) using an ultramicrotome (RMC MTXL; Tucson, AZ, USA), stained with lead citrate and uranyl acetate and observed with a JEM-100 CX transmission electron microscope (JEOL, Tokyo, Japan).

### 3-(4,5-Dimethylthiazol-2-yl)-2,5-diphenyl tetrazolium bromide (MTT) assay

MTT solution (1 mg/ml) was administrated and cells were incubated for 1 h. At the end of the incubation period, 150 μl of dimethyl sulfoxide was added to each well. The amount of formazan crystals formed by the viable cells was determined using a spectrophotometer at 540 nm (GE Healthcare, Piscataway, NJ, USA).

### Flow cytometry

Flow cytometry was performed using a FACS Calibur flow cytometer (BD Biosciences, San Jose, CA, USA). The cells were incubated with annexin V and propidium iodide (PI). Cell damage was assessed with flow cytometric evaluation of cells stained by annexin V and PI, and the data were analyzed using Cell Quest v3.3 software.

### Western blot analysis

Proteins of total cell lysates were separated using SDS-PAGE and transferred to nitrocellulose membranes. The membranes were incubated with primary antibodies, followed by incubation with an appropriate secondary antibody. The following primary antibodies were used in this study: anti-EGFR, anti-phospho-AKT, anti-AKT, anti-phospho-ERK1/2, anti-ERK1/2, anti-phospho-mTOR, anti-mTOR, anti-cleaved caspase-3 and anti-FAK antibodies (Cell Signaling Technology, Danvers, MA, USA), anti-phospho-FAK and anti-β-actin antibodies (Sigma, St. Louis, MO, US), anti-HSP70 and anti-HSP90 antibodies (Abcam, Cambridge, MA, USA), and Ki-67 antibody (Santa Cruz Biotechnology, Santa Cruz, CA, USA). The relative intensity of the bands observed by western blotting was analyzed using the Image J program.

### Xenograft tumor model

Female BALB/c nude mice (5-6 weeks old; Orient Bio, Sungnam, Korea) were housed in the animal care facility of the Biomedical Research Institute of Seoul National University Hospital. Animal care and experimental procedures were carried out in accordance with guidelines on the ethical use of animals that were approved by the Institutional Animal Care and Use Committee (IACUC) of Seoul National University Hospital (12-0353-C2A0). 1×10^6^ viable cells were injected into the right fat pad of the 4th mammary gland. Tumor volume was measured with digital calipers and US imaging using a modified ellipsoidal formula for volume (volume = 1/2(length×width^2^)) [35]. Mice injected with MDA-MB-231-Luc cells were randomly assigned to 4 groups: saline (control) (n=5); anti-EGFR-GN (n=5); anti-EGFR-GN+NIR-PTT (n=5); anti-EGFR (n=5). Mice injected with MCF-7 cells were assigned 2 groups: GN (n=5); and anti-EGFR-GN (n=5).

### NIR-PTT

Heat was applied using a wIRA (Hydrosun model 750, Hydrosun Medizintechnik GmbH, Müllheim, Germany) with a 750 W halogen lamp and a 780 nm high pass filter, yielding a peak output at 820 nm. *In vitro* NIR-PTT (at 1.5 W/cm^2^ for 3 min) was performed in cultured cells at 24 h after treatment with anti-EGFR, GN or anti-EGFR-GN. Based on our previous study [22], we decided the NIR-PTT time point at which anti-EGFR-GNs were maximally accumulated into the tumors of mice after intravenous injection of anti-EGFR-GNs using serial follow-up PAI. When the average tumor volume was 150-200 mm^3^, a circular mask 1.5 cm in diameter was placed over the abdomen to confine the radiation to the tumor region and *in vivo* NIR-PTT (at 1.5 W/cm2 for 3 min) at 48 h after intravenous injection of anti-EGFR-GNs (100 nM, final 0.5 pmol/g mouse) at 3 d-intervals was performed three times. The temperature of culture medium and tumor skin was measured using the non-contact infrared thermometer gun (BENETECH, Shenzhen, China).

### US-guided PAI and bioluminescence imaging (BLI)

US-guided PAI was performed in B mode and PA mode using a preclinical Vevo2100 LAZR imaging system (FUJIFILM VisualSonics Inc., Toronto, Ontario, Canada) equipped with a 40 MHz, linear array transducer. The laser was tuned to optical wavelengths from 750 to 850 nm with a PA signal gain of 40 dB. The relative PA signal amplitude on image slices of tumor was quantified using the post-processing software tools (FUJIFILM VisualSonics Inc., Toronto, Ontario, Canada) [36]. *In vivo* BLI after intraperitoneal injection of D-luciferin (150 ng/kg, Promega, San Luis Obispo, CA, USA) was conducted on the IVIS luminal II system (Caliper, Hopkinton, MA, USA). The sum of all detected photon counts within tumor was quantified in units of mean photons per second per centimeter squared per steradian (p/s/cm^2^/sr).

### Histological analysis

The tumor, liver, spleen and kidney were removed, fixed with 10% buffered formalin and embedded in paraffin blocks. Hematoxylin and eosin (H&E) staining, immune gold-silver staining (Sigma, St. Louis, MO USA) and immunostaining for cleaved caspase-3, Ki-67 and EGFR were performed. Terminal uridine deoxynucleotidyl transferase-mediated dUTP nick end labeling (TUNEL) staining with the ApopTag Peroxidase *In Situ* Apoptosis Detection Kit (S7100, Millipore Ltd., Darmstadt, Germany) was used to detect apoptotic cells. Histological images of stained tissues were acquired using a microscope (Leica, Wetzlar, Germany) equipped with a CCD camera.

### Statistical analyses

All experiments were expressed as the means±standard errors calculated from at least five independent experiments. The ANOVA was performed. A *P* value less than 0.05 was considered statistically significant.

## SUPPLEMENTARY MATERIALS FIGURES



## Abbreviations

NIR-PTT: Near-infrared photothermal therapy
PAI: Photoacoustic imaging
TNBC: Triple negative breast cancer
GN: Gold nanorod
EGFR: Epidermal growth factor receptor
anti-EGFR-GN: anti-EGFR antibody-conjugated gold nanorod
