# Supplementary material for: Near-infrared photothermal therapy using anti-EGFR-gold nanorod conjugates for triple negative breast cancer
Source: Oncotarget. 2017 Sep 23;8(49):86566–75. doi: 10.18632/oncotarget.21243 (PMC5689707; doi:10.18632/oncotarget.21243)
Supplement: Supplementary file 1 [file oncotarget-08-86566-s001.pdf]

Near-infrared photothermal therapy using anti-EGFR-gold nanorod conjugates for triple negative breast cancer

SUPPLYMENTARY MATERIALS

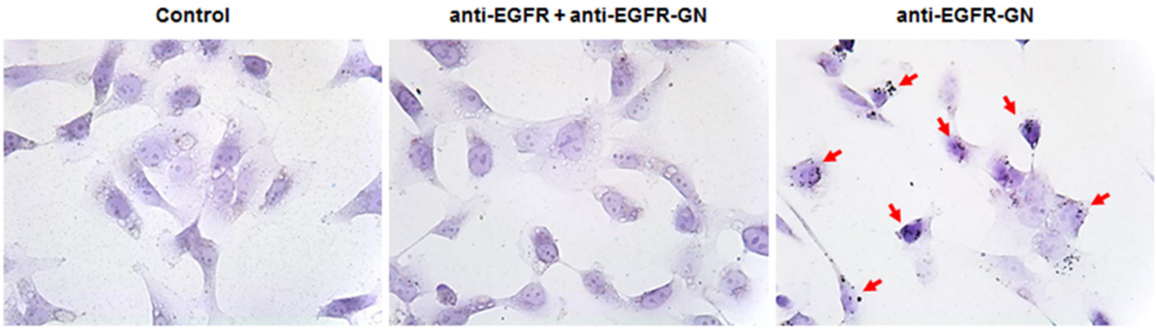

Supplementary Figure 1: *In vitro* competitive inhibition study for analyzing the specific anti-EGFR-GN targeting. MDA-MB-231 cells were pre-incubated with free anti-EGFR antibody (0.22 µg/ml) for 6 h, subsequently treated with anti-EGFR-GN (1.84 µg/ml GN) for 20 h. The specific anti-EGFR-GN targeting was assessed using silver enhancement staining. Red arrows indicate the accumulated anti-EGFR-GNs.

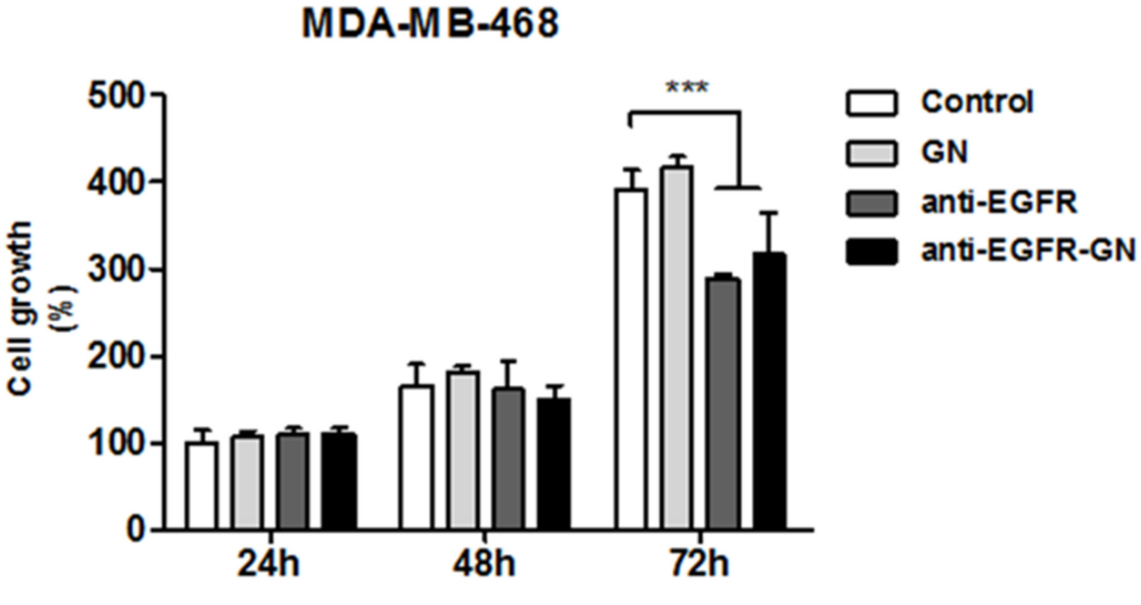

Supplementary Figure 2: Anti-proliferative action caused by anti-EGFR-GN in MDA-MB-468 cells. Analysis of cell growth (mean±S.E., n=3) assessed by MTT assay in MDA-MB-648 cells treated with 1.84 µg/ml of GN and anti-EGFR-GN or 0.22 µg/ml of anti-EGFR antibody for 24 h, 48 h and 72 h.

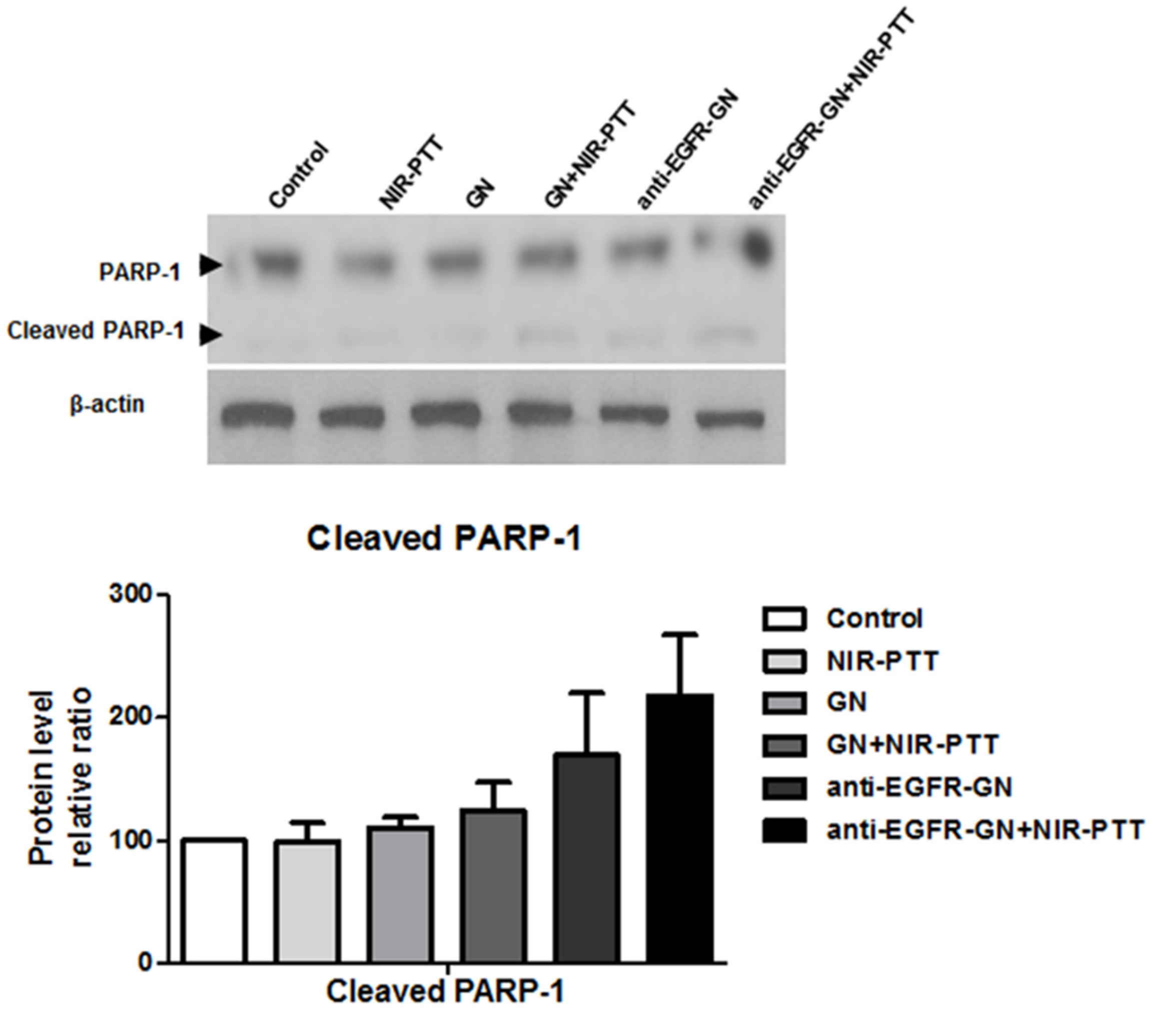

Supplementary Figure 3: Effect of anti-EGFR-GN alone or combination with NIR-PTT on cleaved PARP-1. Western blot analysis of PARP-1 in MDA-MB-231 cells treated with 1.84 μg/ml of GN or anti-EGFR-GN for 24 h and subsequent NIR-PTT for 3 min.

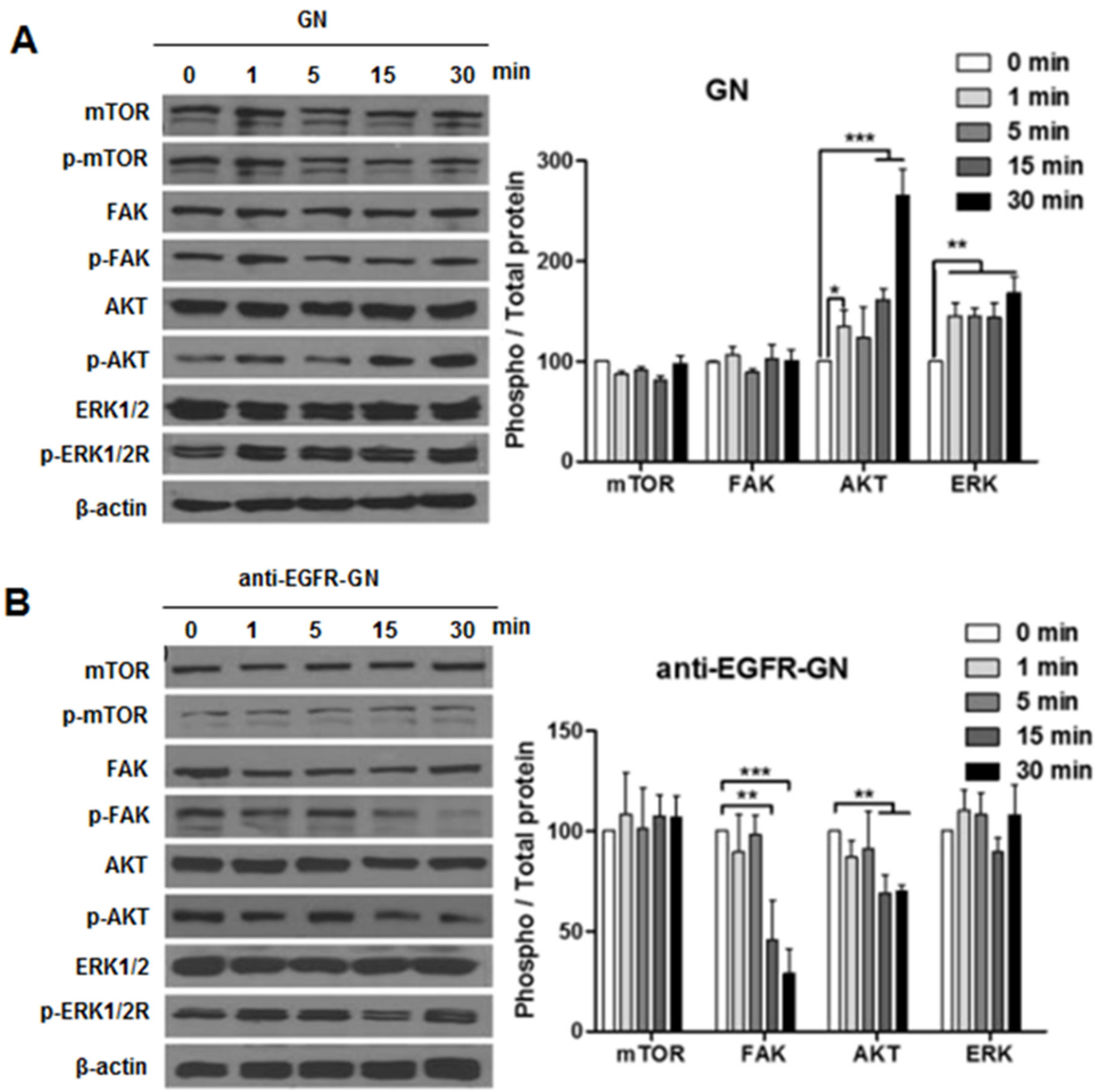

Supplementary Figure 4: Effect of GN and anti-EGFR-GN on phosphorylated mTOR, FAK, AKT, and ERK1/2. Western blot analysis of phosphorylated mTOR, FAK, AKT and ERK1/2 in MDA-MB-231 cells treated with 1.84 μg/ml of GN or anti-EGFR-GN for 1 min, 5 min, 15 min, and 30 min.
